# Co-creation of a health education program for improving the uptake of HIV self-testing among men in Rwanda: nominal group technique

**DOI:** 10.1016/j.heliyon.2020.e05378

**Published:** 2020-10-30

**Authors:** Tafadzwa Dzinamarira, Augustin Mulindabigwi, Tivani Phosa Mashamba-Thompson

**Affiliations:** a Department of Public Health Medicine, School of Nursing and Public Health, University of KwaZulu-Natal, Durban, 4001, South Africa; b HIV/AIDS and STIs Division, Rwanda Biomedical Center, Kigali, Rwanda; c CIHR Canadian HIV Trials Network, Vancouver, BC, Canada; d Department of Public Health, University of Limpopo, Polokwane, Limpopo Province, South Africa

**Keywords:** Public health, Epidemiology, Infectious disease, Clinical research, Health education program, Men, HIV self-Testing

## Abstract

**Objective:**

This study sought to collaborate with key stakeholders to reach a consensus regarding the predominant barriers preventing the uptake of HIV testing services (HTS) by men and co-create an acceptable educational program to improve the knowledge of HIV self-testing (HIVST) among men in Rwanda.

**Methods:**

We employed the nominal group technique to identify a consensus regarding the predominant barriers currently impeding the male uptake of HTS. The health education program content was guided by the ranked barriers. We applied Mezirow's Transformational Learning Theory for curriculum development.

**Results:**

Eleven key barriers currently impeding the male uptake of HTS were identified in the nominal group process. The stakeholders co-created an interactive, structured curriculum containing information on the health locus of control; HIV etiology, transmission, diagnosis, status disclosure benefits, care and treatment services; and an overview of the HIVST background and test procedure to address multiple barriers.

**Conclusion:**

Key stakeholders co-created a comprehensive health education program tailored to men, which integrates education about health beliefs, HIV/AIDS and HIVST. Further studies to assess the effectiveness of the program are needed. It is anticipated that the intervention will improve the uptake of HIVST among men in Kigali, Rwanda.

## 1 Introduction

The Rwandan national HIV/AIDS control program has made significant progress in HIV prevention efforts through established HIV counseling and testing services and the decentralization of the ART provision [1, 2, 3]. However, the number of new HIV infections annually remains a public health concern [4]. A recent national survey revealed 5,400 new infections annually [4]. The majority of new HIV infections in Rwanda are the result of heterosexual transmission in the adult population [5]. Rwanda's HIV response strategy has heavily relied on the well-established implementation of voluntary HIV counseling and testing (VCT) [6, 7] and provider-initiated HIV testing services (PITC) [8, 9] nationwide. Results from the Rwanda Population-based HIV Impact Assessment (RPHIA) show that 76 percent of all HIV-positive adults, including almost 80 percent of HIV-positive women, have achieved viral load suppression, a widely used measure of effective HIV treatment in a population. This surpasses the Joint United Nations Programme on HIV/AIDS (UNAIDS) target of 73 percent by 2020. Rwanda has made tremendous progress by reaching or exceeding the UNAIDS 90–90–90 targets particularly among women and, nationally, by attaining 84–98–90 among adults [4]. Despite the major success reported for VCT in PITC, there remains a significant unmet need for HIV testing services (HTS) among men [4, 10]. Data from the 2015 Rwanda Demographic and Health Survey [10] and a 2019 national cross sectional survey [4] revealed that 24% and 20% men were unaware of their HIV status, respectively.

Evidence gathered from a study conducted in six sub-Saharan African countries revealed that men tend to report less frequent HIV testing than women [11]. This gap is seen across Africa, with multiple studies reporting lower levels of HTS uptake among men compared to those among women [12, 13, 14, 15, 16]. Yamanis *et al.* recommended making HIV testing appear more normal as a strategy to improve the uptake of HTS among men [17]. Further, a number of qualitative studies have revealed a greater acceptability of home-testing or self-testing than facility-based testing among adolescent [18, 19] and adult men [20, 21, 22].

HIV self-testing (HIVST) is a relatively new strategy, recommended by the World Health Organization (WHO) [23, 24], which has been successful in increasing the testing uptake among underserved populations, including men [25, 26, 27, 28, 29]. A cluster-randomized trial in Malawi reported an increase in the proportion of outpatients tested for HIV in the HIVST intervention group, compared to the PITC groups (adjusted odds ratio 8·52, 95% CI 3·98–18·24) [30]. The procedure for HIVST involves individuals collecting their own blood or oral fluid specimen, conducting a rapid diagnostic HIV test, and interpreting the result [24]. Pro-HIVST arguments among men in a United States study were that HIVST is a good addition to facility-based testing, offers privacy and convenience, does not require counselling, and could lead to a linkage to care [31]. However, they also had concerns regarding the accuracy of HIV self-tests, their cost, and receiving a positive test result without immediate access to follow-up services [31]. Available studies from Eastern Africa have shown HIVST to be acceptable among men [28, 32, 33, 34, 35, 36].

Health education programs have been reported to improve health outcomes among various groups of people [37]. For instance, a sexual and reproductive health curriculum among the youth [38] and post-operative patients with breast cancer [39] resulted in an improved health status. In Nigeria, health education programs delivered to antenatal care attendees with malaria [40] and HIV positive individuals with tuberculosis [41] resulted in an improved knowledge, attitude and practice. Health education programs for sexual and reproductive health implemented in Uganda [42, 43] and Kenya [44] have resulted in an improved health-seeking behavior among men. In the context of HIV, health education resulted in a reduced HIV risk behavior among migrant sex workers [45]. In a separate study, health education increased the HIV knowledge and HIV/AIDS preventative behavior among household wives [46]. To our knowledge, there is a paucity of evidence on the effectiveness of health education programs tailored to men in the context of HIV.

Rwanda officially introduced HIVST on World AIDS Day in 2017 [47]. HIVST guidelines were recently included in the national HIV prevention and management guidelines on July 1, 2018 [48]. Qualitative studies conducted in Rwanda among men [36] and key stakeholders [49] have reported poor knowledge as an important barrier preventing the uptake of HIVST among men. However, none of these studies have explored potential strategies to address the barriers. Therefore, it is important to provide HIVST health education to men to maximize their uptake and improve their health outcomes. It is also recommended that ` stakeholders are involved in developing and adapting HIVST health education programs. Collectively, with key stakeholders, we sought to develop an educational program that can be employed to improve knowledge of HIVST among men in Rwanda. In this study, we defined key stakeholders as people who are likely to have expertise knowledge on HIV services, men's health services, HIVST and health promotion in Kigali, Rwanda and men who resided in Kigali at the time of the study, representing the target population.

## 2 Methods

### 2.1 Setting

Rwanda is a country located in the Eastern African region, with an estimated population of 11.5 million [50]. The country has the highest population density in Africa of 467 people per km^2^, with more than 80% of the population residing in rural areas [51]. The HIV prevalence in the general population in Rwanda is 3% [4]. The prevalence is higher in females (3.7%) than in males (2.2%) [4]. Kigali City Province is the capital city of Rwanda. The HIV prevalence in Kigali City is 4.3% [4]. The prevalence of HIV increases with age and is higher among women compared to their male counterparts [4, 10]. Kigali City houses all national-level stakeholders in the HIV program in Rwanda [52]. The study was conducted in Kigali City at a venue convenient for a research workshop.

### 2.2 Study design

This study was part of a multi-phase study aimed at the adaptation of a health education program for improving men's uptake of HIVST in Kigali, Rwanda. The protocol for the main study is published elsewhere [53]. The main study is a mixed method study, conducted in four phases. In phase 1, we conducted a scoping review to map the available evidence on health education programs for men in LMICs [37] and a narrative literature review on the factors contributing to men's engagement with HIV services [54]. In phase 2, we conducted interviews with stakeholders in the Rwanda HIV response to determine their perspectives on the implementation of HIVST in Rwanda [49]. In phase 3, we employed a cross sectional survey to assess the HIVST awareness and acceptability among men in Rwanda [55]. The research questions for the current study were guided by the findings of the qualitative study in phase 2 [49]. Guided by the findings in phase 1, 2 and 3, the current study employed the nominal group technique [56] to map out a consensus on the predominant barriers preventing the uptake of HTS among men in Rwanda and co-create a health education program (HEP) for improving the uptake of HIVST among men in Rwanda. The HEP will be administered to men in a hospital setting by trained health professionals. The current study findings inform the next phase of the main protocol; a randomized control trial to assess preliminary effectiveness of the HEP [53].

### 2.3 Selection and recruitment of participants

We invited key stakeholders in HIV services to participate in a co-creation workshop. Individuals were selected based on the researchers' assessment that they would contribute valuable insights regarding the barriers currently preventing the male uptake of HTS and strategies for the implementation of HIVST to address these barriers. We recruited study participants using the snowball sampling technique. In this study, the term “key stakeholder” will be used to represent a group of subject matter experts (SMEs) and representatives of our target population (the potential users of HIVST). We defined SMEs as people who are likely to have expert knowledge on HIV services, men's health services, HIVST and health promotion in Kigali, Rwanda. An initial list of SMEs was provided by the Division of HIV, Rwanda Biomedical Center. These individuals were approached by the researcher and invited to participate in the workshop. In cases were an individual was unavailable or felt someone else would be better placed to participate in the study, snowballing method was used to invite the second individual. We defined our target population representatives as men who resided in Kigali at the time of the workshop. All participants spoke Kinyarwanda.

### 2.4 Nominal group process

We employed the nominal group technique [57] method to conduct a highly structured group discussion to attain a group consensus on the priorities in response to our specific research questions. We conducted the workshop in two phases:

Phase 1: Consensus on the prioritization of barriers that currently impede the male uptake of HIV testing services.

Phase 2: Co-creation of a health education program that addresses the barriers and aims to improve knowledge on HIVST.

We created 4 sub-groups of 3 participants, ensuring that each group contained one representative of the target population and 2 SMEs. The Principal Investigator (first author) served as the convener and moderator for the group. In the first step, the participants independently listed their response to the research question. Second, in a round-robin fashion, each participant presented one idea at a time to their group [56, 57]. Thereafter, one representative of each sub-group presented to the main group and the ideas were recorded verbatim [56]. The groups then discussed and clarified the responses. At this stage, the responses were grouped together into themes [56]. This session was convened, until we reached data saturation (i.e., additional groups no longer elicited new concepts). In the final step, ideas were ranked and prioritized [58]. In the final step, items were ranked from least to most severe barriers to HTS uptake. The ranking scores were between one and five, one being the least severe, and five being the most severe current barrier to the uptake of HTS.

### 2.5 Development of the health education program

The development of a HEP was informed by the Mezirow's Transformational Learning Theory [59]. According to Mezirow's philosophical approach, the humanistic assumption would mean that men (recipients), as humans, need to rethink their actions deeply in order for them to act with more insight and effectiveness [59]. Men can rethink and reflect on those barriers that prevent them from taking up HIVST. The humanistic approach further presupposes that men have an opportunity to play a significant role in their health care with regard to the uptake of HIVST.

Based on the ranked barriers, all stakeholders proposed key messages to include in the health education program to address each theme. SMEs proposed the content structure of the health education program, while the target population representatives validated the potential users' comprehension. The content of the curriculum was validated by the HIV Division, Rwanda Biomedical Center for alignment with current policies, guidelines and regulations.

### 2.6 Data management and analysis

To obtain the quantitative data, which were gathered during the ranking step in the nominal group process, a total importance score for each barrier was calculated by summing the individual scores of the participants. We analyzed the qualitative data using thematic content analysis to inductively identify the themes that emerged from the data presented during the discussion. The data analysis was based on the naturalistic paradigm, with a conventional content analysis [60], in which coding categories were derived directly from the text data. This approach has been shown to limit researcher biases due to preconceived ideas or other theoretical perspectives [61]. The first and second author performed the data analysis.

### 2.7 Ethics

This study was ethically reviewed and approved by four institutional review boards: Rwanda National Ethics Committee (Approval number: 332/RNEC/201), University Teaching Hospital of Kigali Ethics Committee (Approval number: EC/CHUK/0111/2019), Rwanda Military Hospital Institutional Review Board (Approval number: RMH IRB/036/2019) and the University of KwaZulu Natal Biomedical Research Ethics Committee (Approval number: BE/280/19). All participants were provided an information sheet explaining the study background, objectives and procedures. Signed consent was obtained, prior to any study procedures.

## 3 Results

### 3.1 Participants

Eight SMEs and 4 representatives of the target population aged 30–56 agreed to participate in our workshop. Of these, 5 (42%) were male. The majority (92%) of the study participants were employed, with only one unemployed. Of the SMEs, 2 represented the HIV Division, Rwanda Biomedical Center, 3 were from primary health facilities working in HIV and social work services, 2 were from non-governmental organizations working on HIV in Rwanda, and 1 was from Rwanda's largest referral hospital. The characteristics of the participants are presented in Table 1 below.

**Table 1:** Characteristics of the workshop participants.

ID	Gender	Age	Highest Qualification	Title	Designation
1	Male	30	Master's degree	Quality Assurance and Control Manager	Target population representative
2	Male	38	Bachelor's degree	Business Manager	Target population representative
3	Male	56	Secondary 6 diploma	Community Health Worker	Target population representative
4	Male	55	Secondary 3 certificate	Head of Village*, Umutako*	Target population representative
5	Male	36	Master's degree	Prevention Programs Manager	SME
6	Female	36	Bachelor's degree	Nurse in charge VCT/PMTCT	SME
7	Female	40	Bachelor's degree	Nurse mentor/HIV Services	SME
8	Female	48	Bachelor's degree	In Charge of Social Work/HIV Services	SME
9	Female	40	Secondary 6 diploma	In Charge of HIV Services	SME
10	Female	46	Bachelor's degree	In Charge of VCT	SME
11	Female	48	Master's degree	Ag. Director of HIV Prevention Unit	SME
12	Female	46	Master's degree	VCT Senior Officer	SME

### 3.2 Stakeholders' perspective on the barriers currently preventing the uptake of HTS among men in Kigali, Rwanda

**Figure 1:**
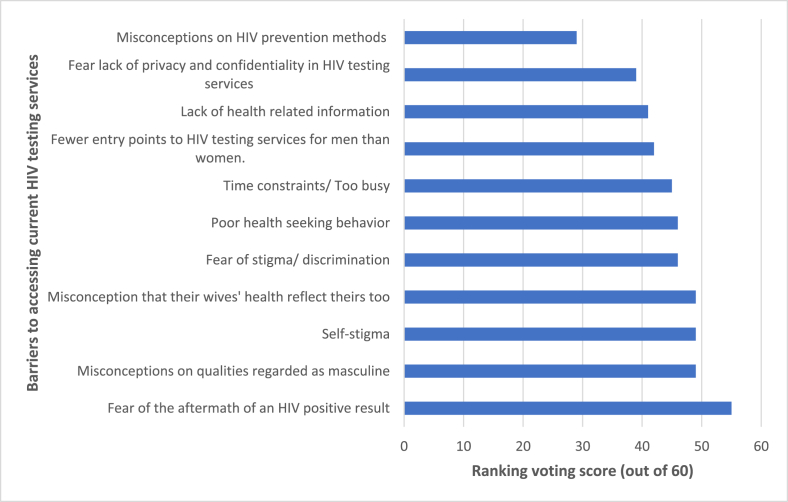
Key stakeholders' voting scores for barriers preventing men from accessing current facility-based HIV testing services in Kigali, Rwanda.

The stakeholders reported eleven factors as barriers preventing men from accessing current facility-based HIV testing services in Rwanda. Supplementary File 1 presents the qualitative data. Figure 1 presents the ranking results. The voting results showed that fear of the aftermath of a HIV positive result is the most severe barrier, followed by misconceptions regarding the qualities regarded as masculine, then self-stigma and misconceptions that their wives' health reflect theirs. Misconceptions regarding HIV prevention methods was voted as the least severe barrier, followed by fear of a lack of privacy and confidentiality.

### 3.3 Health education program to improve the uptake of HIVST among men

**Figure 2:**
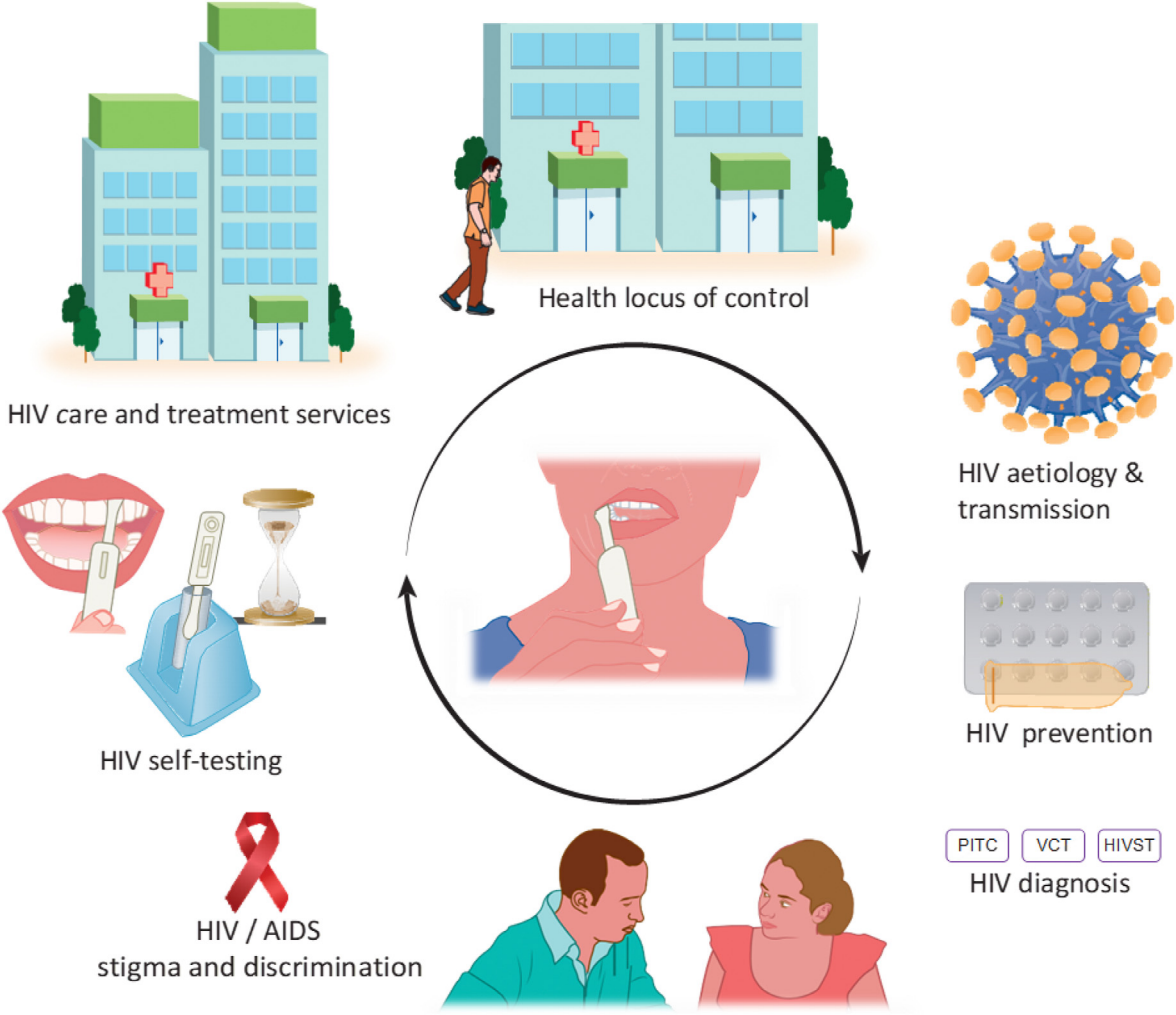
Health education program summary.

From the guided barriers revealed by stakeholders, we designed a male-tailored curriculum to help improve the uptake of HIVST among men in Rwanda (Table 2). This curriculum comprises a range of modules targeted at men in Rwanda. The curriculum will be administered in both English and Kinyarwanda. It is estimated that this curriculum can be taught in an hour. The curriculum will include the following key concepts: The health locus of control and HIV etiology, transmission, diagnosis, treatment, status disclosure, stigma, discrimination, and care and treatment services. It will also cover the background and test procedure of HIVST. The health locus of control is defined as the degree to which people believe that they, as opposed to external forces (beyond their control), have control over their health outcomes [62]. An internal health locus of control suggests that positive health results from one's own doing, will power or sustained efforts [62]. The motivation to control one's health may predict health behavior [63, 64]. The full content of the HEP is outlined in Supplementary File 2 and is summarized in Figure 2. Representatives of the target group validated all curriculum contents and proposed appropriate message packaging approached in order to increase the likelihood of acceptance when offered to men.

**Table 2:** Health education program content to address the identified barriers preventing the uptake of HIV testing services among men.

Themes	Program content to address theme
Fear of the aftermath of an HIV positive result	- HIV care and treatment services summary - HIV status disclosure benefits
Misconceptions concerning the qualities regarded as masculine	- Health locus of control discussion
Self-stigma	- Health locus of control measure statements - HIV care and treatment services summary - HIV status disclosure benefits
Misconception that their wives' health reflects theirs too	- Health locus of control discussion - HIV etiology and transmission
Fear of stigma/discrimination	- HIVST current distribution methods - HIV care and treatment services summary - HIV status disclosure benefits
Poor health seeking behavior	- Health locus of control measure statements
Time constraints/Too busy	- Health locus of control discussion - HIVST current distribution methods - HIVST procedure
Fewer entry points to HIV testing services for men than women	- HIVST current distribution methods
Lack of health-related information	- Health locus of control discussion - HIV etiology and transmission - HIV care and treatment services summary - HIV status disclosure benefits
Fear of a lack of privacy and confidentiality in HIV testing services	- HIVST current distribution methods
Misconceptions concerning HIV prevention methods	- HIV etiology and transmission

## 4 Discussion and conclusion

### 4.1 Discussion

This study presents the consensus of HIVST key stakeholders on the content of HEP for improving the uptake of HIVST among men in Rwanda. The provision of health education to improve the uptake of HTS among men is underscored in both national [48] and international [24] recommendations. The stakeholders reported multifaceted barriers to the uptake of HTS among men and proposed an equally comprehensive and integrated curriculum to address these barriers.

Fear of the aftermath of a positive status result was ranked as the top priority barrier in this study. This corroborates the findings of a qualitative study conducted among male health care providers in South Africa [65], which revealed fear of a loss of material resources through lost employment and rejection from family and community as barriers [65, 66]. Our findings revealed stigma to be an important barrier preventing the uptake of HTS among men. Similar findings were reported in a study conducted among male urban commuters in South Africa [67]. Fear of discrimination emerged as an important barrier to the uptake of HTS among men in this study. Similar findings were reported in a recent review paper [68]. Sullivan *et al.* reviewed papers published between 2014 and 2019 from Kenya, Malawi, Mozambique, Nigeria, South Africa, Tanzania, Uganda, Zambia and Zimbabwe. Men who have sex with men in Nigeria reported fear, public harassment, and the experience of sexual violence as barriers preventing them from seeking HTS [69]. These findings underscore the need to further strengthen differentiated delivery service models that attract men such as private pharmacies. The current study revealed time constrains as a major barrier to the uptake of current facility-based HTS among men. This corroborates findings in Malawi, where rural men reported that experience of going to health facilities was the major factor that deters men from testing [21]. Interestingly, men have reported concerns that they may be perceived as less masculine if they sought health services from primary health clinics in South Africa [65, 66, 70], Congo, Mozambique, Nigeria and Uganda [71, 72].

The current study has recommended a discussion on the health locus of control with men as a potential strategy to improve their health seeking behavior and ultimately improve their uptake of HIVST. The health locus of control was one of the parameters of health belief used in designing health education programs for college students [73]. A 5-country group health education intervention, aimed at reducing HIV risk behavior, employed discussions on individuals having control over their health outcomes [74]. The current study also recommended an overview of the disease as a key content for a curriculum to improve the uptake of a testing methodology of this disease. This approach has been used elsewhere for the uptake of tuberculosis testing [41], HIV VCT in Nigeria [75], HIV awareness in China [76] and PaP smears for cervical cancer in Ghana [77]. Modules on sexually transmitted diseases and HIV were incorporated in a health education program in South Africa, developed for men who had undergone initiation and traditional male circumcision [78]. Similarly, multiple health education curricula for HIV/AIDS risk reduction programs among youth in Africa adopted a similar approach [79, 80, 81].

A notable strength of the current study is the inclusion of the target population in the sample, as this critical population may have inherently different perspectives on the composition and structure of the curriculum from those of the SMEs. The major benefit of using NGT is that the stakeholders can reach a consensus. In this study, the stakeholders managed to build a consensus on the predominant barriers preventing the uptake of HTS among men to guide the co-creation of an educational program. NGT allowed the researchers to obtain qualitative data in an open-ended manner, which were subsequently quantified by ranking. The themes were not selected a priori but rather actively constructed by the group. Future researchers may replicate the methods to design curricula to address various research problems in public health. On the other hand, a limitation of this study is the gender composition of the stakeholders. The majority (7) of the SMEs enrolled were women, thus limiting the generalization of the study findings. However, we feel that they were selected on the basis of their expertise and provided valuable insights into the research questions. A second limitation of the study is the point that all 4 representatives of the target population were educated. However, we feel it was important for the representatives to have some form of education to allow for a written input and validation of the HEP content. Finally, the group consensus achieved is rather limited to the local context due to the lack of a larger scale of representative survey, limiting generalizability for the entire country, Rwanda.

### 4.2 Conclusion

Key stakeholders identified socio-behavioral and socio-cultural factors as predominant barriers preventing the uptake of HTS among men. In response, key stakeholders co-created a comprehensive health education program tailored to men, which integrates education about health beliefs, HIV/AIDS and HIVST. It is anticipated that the intervention will improve the uptake of HIVST among men in Kigali, Rwanda. At the time of writing this article, the curriculum is currently being piloted at a teaching hospital in Kigali on men attending the facility as clients for health services or for other reasons.

## Data availability

The transcripts from the qualitative component of this study are available on Supplementary File 2.

## Declarations

### Author contribution statement

T. Dzinamarira: Conceived and designed the experiments; Performed the experiments; Analyzed and interpreted the data; Wrote the paper.

A. Mulindabigwi: Performed the experiments; Analyzed and interpreted the data; Wrote the paper.

T. Mashamba Thompson: Conceived and designed the experiments; Wrote the paper.

### Funding statement

This work was supported by 10.13039/501100004695Inyuvesi Yakwazulu-Natali (641581).

### Declaration of interests statement

The authors declare no conflict of interest.

### Additional information

No additional information is available for this paper.

## Appendix A Supplementary data

Supplementary File 1 Interview transcripts Supplementary File 1 Interview transcripts

Supplementary File 2 Full contents of HEP Supplementary File 2 Full contents of HEP
