# Supplementary material for: Co-creation of a health education program for improving the uptake of HIV self-testing among men in Rwanda: nominal group technique
Source: Heliyon. 2020 Oct 30;6(10):e05378. doi: 10.1016/j.heliyon.2020.e05378 (PMC7610321; doi:10.1016/j.heliyon.2020.e05378)
Supplement: Supplementary File 1 Interview transcripts [file mmc1.docx]

Co-creation of a health education program for improving the uptake of HIV self-testing among men in Rwanda: Nominal group technique

Tafadzwa Dzinamarira, Augustin Mulindabigwi and Tivani Phosa Mashamba-Thompson

**Supplementary File 2 (Interview transcripts)**

1. **Groups presentation on barriers impeding men from seeking HIV testing services**

**[Group 1 reported barriers]**

**Moderator:**  If group one is ready, we can welcome them to present their shared ideas in 10 minutes. Remember every group has 10 minutes to present to us the barriers that they agreed on together, but if any one gets a new idea that was not discussed, they can comment or write it down on the sticky notes. Group one you can start please

**Male Speaker 1:** The first barrier we discussed in our group is self-stigma. Men feel like how would people at the HF look at me I am on the line for ST! men think that people will be judging them and think that if he came to the clinic for testing it is because he did something [sex] or he is suspicious. We made a comparison to when it comes to men and women at the HC. Women can take the opportunity of coming with babies and also use the ST services and no one will try to think more about their reason to visit the services while if a man enters, he thinks everyone knows why he is there. The second point is that men are resistant in their nature.

**Male speaker 2:** But if I can add on that, come on, we are all mature. Men are in nature tempted to have multiple sexual partners or mess up. So, you can imagine a man who already thinks that he might be exposed. Trust me he won’t be going for a checkup because he is already worried of the results.

**Male speaker 1:** Men also do not get access to health-related information. They are those sketches passing …. Those stuffs, depending on where they happened from. So, men do not listen or follow the talking that happen in the cell levels or teaching shows on radios. We meant that they are normally not aware of the played sketches because of the perception that they are meant for kids and women. There is no way a man like me can sit with the kids and their mother and start listening to the plays

**Moderator:** Do we have examples of the played sketches?

**Male Speaker 2**: like Mashirika, urunana….

**Female speaker 1**: and Seburikoko [laugh]

**Male speaker 2:** yes. There is no one single man who listens to them [sketch plays]. I can be a simple example, when I reach home after work and those sketches are playing, I always change the channel and want to switch to news and football channels. And let’s remember that that it is through those channels that we don’t pay attention that the government uses to deliver health-related messages. So, men don’t have the opportunity to receive

**Moderator:** Let’s all get on the same page hear, is it low access to information or is there any another particular reason?

**Female speaker 1:** It’s not the matter of access, men are not just not interested in those messages from the radio or TV taking or informing the public on health.

**Male speaker 1:** but no access results. Or rather the end consequence is that men end up with no information.

**Female speaker 2:** But I am not sure if a person in another group can comment on that?

**Moderator:** there is no problem please go ahead

**Female speaker 2:** There are two things that the first speaker mentioned but I think there should be involvement of other stakeholders. For instance, that issue he raised of men having multiple sexual partners and end fearing the testing process, why do we think that it is only men who should go through that. Women should also be scared because those men don’t have sex themselves, I mean women are fully involved and enjoying [laugh]. Another comment is on the sketches that he mentioned. Of course, a man comes hope and he all he wants is to change the channel to news and football. But how about that wife and daughter who followed the health promotion and education sketches, this is where their involvement could show so that they help to inform the men on what they leant

**Male Speaker 1:** Still remember the second point we made on resistance. Men would not sit there and listen to people at home teaching them about health. All he wants is to watch his match and relax [inaudible 4:52] another thing is that men fear a positive result. What if I find I am HIV positive, what do I tell my kids and mostly my wife?

Moderator: okay. But are men to fear a positive result and not women as you are saying?

**Male speaker 2:** Listen, we also asked ourselves that question in our group. We found that a man at his home is considered as a God! A man to think that his kids and wife will find out his HIV status, according to him that is shameful, they don’t like that shame.

**Male speaker 1:** So that fear that keeps going on and on, already it is a barrier because he keeps pushing his testing appointments everyday and it ends up him not going to the clinic at all.

**Moderator:** so that man wouldn’t wish for anyone to know his status, right?

**All**: yeah

**Male Speaker 1**: Another barrier then is the usual that we all have heard of. A man believes that if my wife has gotten her HIV results from the clinic, then that’s it. Luckily if she got a negative one then I am also not infected.

**Sharon:** Do we have a specific term for that misconception?

**Female speaker 2:** It may be lack of information

**Moderator:** not really

**Speaker 1:** I am not sure what the theme there should be

**Moderator:** maybe to remember the scenario, that man will say to himself that if my wife tests regularly and finds no challenge in doing so, therefore I will be following her results.

**Male speaker 2:** you are right. And also, majority of men believes that their women will never bring any diseases at home. They always assume that if there is something wrong going on in their relationship or at home, let’s say his wife goes and tests positive, the answer is that a man is behind that.

**Male speaker 1**: Our next barrier is like a moral comparison. We did a moral comparison and found that men do knot have so many ways as their counterpart women that ease their access to self-testing services. You find that women have opportunities to test when they attend ANC, and when they go for the kids’ vaccination or even maternity, they can always test via those ways unlike men who never attend them

**Male speaker 2**: ooh yes even when she didn’t intend to test, she will end up testing

**Female speaker 3**; is it by force then?

**Male speaker 2**: not really by force but there are so many opportunities or let’s say women found themselves in positions to need to know their HIV statuses

**Female speaker 2**: [laugh] but why don’t men accompany their wives to the clinic so that they also profit from those services [laugh]

**Moderator**: Do we all understand that barrier, they just mentioned men having few entry points than women? Men only get tested during VCT or regular OPD

**Male speaker 2**: another entry point for men that we discussed was related to churches like SDA. You find that they are required to provide their HVI statuses in order to be allowed to wed. A guy and a lady have first to show an HIV testing certificate before the church allows them to get married. So young men who are preparing to have their own homes nowadays are using that opportunity to go and test. That’s the only way some men are finding that they cannot dodge the testing exercise

**Female speaker 2:** Is it only the SDA church?

**Male speaker 2:** I guess even some other churches do so but I am well aware and sure of the SDA church

**Moderator:** there is another church where they even ask for the pregnancy test. So, are you saying that all churches now require HIV test results before the wedding?

**Female speaker 1:** No, they just want to know if you have taken the test but they don’t ask them for their HIV statuses

**Moderator:** Okay, that is possible then. I thought they even ask them for their statuses.

**Male speaker 1:** So, the last barrier related to the previous one is privacy-related. All those barriers that we discussed hinders a man his privacy. He doesn’t have a place where he can go and feel like no one is watching or staring. As we said earlier, that man is not taking a child for ANC and then end up also testing so few entry points is a strong barrier

**Male Speaker 2:** On that privacy issue, we tried to give real examples on ourselves. That man there should excuse me but we used him as an example and thought that imagine that man going all the way to the clinic for just an HIV test. So, we thought that how about imitating that intervention that is used among the youths during awareness and sensitization, the so-called youths’ corners. Why not corners specific for just men where they can meet as only men and also have some privacy.

**Male speaker 1:** In a nutshell, those are the barriers that we discussed as group one. Thank you so much

**Moderator:** Thank you so much group one. Let’s all clap for them.

**[Group 2 reported barriers]**

**Female speaker 4:** group members are [mentioning the names]

**Moderator:** can we have all post-it notes here on the flip chart. We will hold onto them and you can present those ideas that your group combined. But remember this is a discussion so if you group members want to add anything, everyone is motivated to interrupt and give new ideas or even comments. feel free, please continue

**Female speaker 4:** In general, most of the barriers just got mentioned by the previous group but we will have to repeat them for the sake of discussion and ideas sharing. We found eight barriers in summary.

**Moderator:** okay

**Female speaker 4:** men have a problem of time and they are impatient. On the time barrier, we tried to imagine that scenario when a man visits the clinic. When he reaches there, and because of all those lab tests he is required to provide, it takes him time and he ends up giving up. His excuses are always related to house or home chores like I left my cow at home and I need to look after kids as if his woman is not around …[laugh]. Arrogance and pride are the second challenges discussed by our group. Men have a tendency of showing off or being too proud. Of course, not all of them but the majority. Ignorance is another barrier because men don’t care about their health status. I remember when I used to work as a hospital receptionist, I could notice the difference between men and women, when it comes to being curious or eve stressed about knowing their health status but men only come for a single test and you dare mention another one, they will say that time does not allow them to wait for the results. Women ask many questions related to their health when they visit the clinic…. [inaudible 20:38] …she will be like doctor please tell me is everything okay, how is my stomach? How about we do more tests. But when men come for checkup, they seem not to care at all even when they come at the last stage of their illness.

**Female speaker 2:** uuuuhm I wanted to add something... sometimes you find that find that they are aware of the news or they have the information but they don’t care. If we can relate that with what she just said on men attending to clinical stuff when their symptoms have reached the last stages of diagnosis and while women do regular check-ups throughout. You notice that women have future worries of their families and this pushes them to know how healthy they are regularly. If we can call this negligence I don’t k now but men have information about regular checkup, it is just they don’t go for one

**Sharon:** it is part of negligence

**Moderator:** sure. There is also one thing that was mentioned, I am just curious about why men come when they are really ill and yet they know HIV exists and other health-related problem.

**Female Speaker 2:** you are right because we mentioned lack of information as a barrier to men accessing early diagnostics. We can relate it to a barrier that was mentioned by the first group about meeting places or entry points. Whenever men meet, you can never hear them discussing about HIV or STIs, they always talk about businesses, cars, while women always discuss health problems. For instance, when a woman tells another one about her husband’s fornication, the advice is to go and do a check up immediately to find out if she hasn’t bene infected already but men don’t have time talk about such intimate secrets therefore no interest or burning feeling for checkup. That is the difference between men and women when it comes to access to information and what do they do after the information. Fearing of positive results and long-life treatment is another barrier.

**Moderator:** How can you relate that barrier of fear of a positive result to that barrier of not attending HTS?

**Female speaker 4:** it is a matter of if I find that I am HIV positive, how will mange that long treatment process

**Moderator:** so, you are saying that they have fear before even getting for the testing?

**Female speaker 4:** yes

**Female speaker 2:** while we are still on that point, I think we can relate this barrier with lack of information where a man thinks that HIV positive equals death, equals my family is gone etc.…

**Female speaker 4:** I see we even repeated the barrier on lack of information sharing between couples or among men. Self-stigma was also a barrier we discussed. Men don’t like that line at the clinic where everyone will know why they visited the HIV department as discussed by our colleagues from group 1

**Male speaker 2:** They discriminate themselves at an early stage. So, what I can add on that is that barrier goes with the arrogance and pride. Normally men we don’t enjoy that long waiting or the lines found at the hospitals to the extent that if a man finds a way of paying more in exchange of a fast service, they are willing to do so. They love shortcuts

**Female speaker4:** The last barrier discussed is behaviour change or mindset. Men are polygamists and do not think it is a problem.

**Moderator:** how is related to what you just discussed?

**Female speaker 4:** you see that masculine side of men causes them to refuse or rather the behavior barrier will always hinder access to HTS because of privacy and all those cross-cutting barriers

**Make speaker 3:** how we explained that barrier of behaviour change or masculinity is like this: a man has desires of a lot of women, while a woman sticks to one man and women don’t speak out on polygamy nature of men

**Moderator:** so, its’s nature or culture?

**Male speaker 3**: it is in their nature for men to have multiple sex partners

**Female speaker 2:** of course, men’s masculine nature is a barrier

**Female speaker 4:** yes. Thank you so much. That is all from group two

**Moderator:** Thank you. And group three the presentation stage is yours. Kindly come with all the sticky notes for our reference

**[Group 3 reported barriers]**

**Female speaker 5:** Group three is also sharing the barriers and we won’t take time as most of them have been repeated several times. Allow me to start to those that were not mentioned. There are a lot of myths with regard to HIV prevention methods like circumcision and condom use that hinder men to HTS. Majority of men who are circumcised believe that that 60% of no risk is not of concern. They believe that circumcision is way of protection and they

**Female speaker 2:** So, it seems that they don’t even understand what that 60% means which is again lack of information. Because if 60%protects you, how about that 40% that remains?

**Female speaker 5:** Another barrier is fear of stigma and discrimination from what their previous exposures.

**Moderator:** okay okay. So, you are saying that men first do a risk assessment before deciding if they will visit HTS or not? And if they think the risk is high, they assume the results they will get will be a positive one. If so, can we relate it to the one of fear of a positive result.

**Female Speaker2:** Allow me to add on that. That fear of a positive result normally goes with their behaviours. For instance, if a man is aware that one of the women, he had sexual relations with is HIV positive, he will automatically assume that he is positive too.

**Female speaker 5:** before I continue I saw one of my group members raising his hand?

Male speaker 4: ooh yeah but she just mentioned it, I want to say that men after judging their behaviour is the reason why they tend to assume that they are already infected even before deciding to visit the clinic for confirmation

**Female speaker 5:** Another barrier that even other groups talked about is that of self-stigma. Where they even went on suggesting some men-only places for privacy. Also, the fear or lack of trust in healthcare providers. They don’t trust confidentiality from healthcare providers. Another one already discussed by other groups is the time constraint. Long time waiting was mentioned.

**Moderator:** Let’s really understand this barrier because it is repeating. Do men really don’t have time to wait in the line as women do or there is another reason behind?

**Female speaker 3:** [laugh] they just make themselves busy and busy again and the one who may have time will always try to be busy [laugh]

**Moderator:** okay…but frankly speaking, is the waiting line real or men just imagine it?

**Female speaker 5:** the long waiting line at health centers is real honestly and if you add that to their impatient and making themselves too busy nature. That is the barrier we are talking about

**Female speaker 2:** But truth be told people, is there a line or long waiting in the VCT services?

**Female speaker 7:** Not at all. I just think men has that perception that there is always a line at the clinics but as you know there are no lines at VCT departments.

**Female speaker 4:** exactly and let’s say on the line it is only two people in front of him, according to him that is still a line [laugh].

**Female speaker 5:** The two last barriers that we discussed as well was that of assuming that their wives’ results are also theirs. If my wife test and is infected, that means I am the one who infected her and that is it. Another one is that men don’t like instructions from healthcare providers. So, we can relate it to their ego nature [laugh]. So, imagine the health care provider is a woman, he won’t feel comfortable to sit and listen to what is going on. We thank you as group three and that is the barriers we discussed.

**Moderator:** Thank you group 3 members. Group 4 you are next please.

**[Group 4 reported barriers]**

**Female speaker7:** Barriers have been mentioned of course but we may have to insist on some. First as someone who works under VCT services, I always notice that the number of women who attend outnumbers that of women because men still believe that their partner’s results reflects theirs too. We need to strengthen health promotion and education so that men are aware of the fact that everyone needs to self-test. This barrier is combined with that one where other group mentioned the fear of their wives. What will I tell my wife or partner if I turn out being positive especially if let’s say he knows he cheated on his partner and when the wife comes and says I am just from the clinic and I have my HVI results they are okay. He will still fear to go and test because he knows his characters of having other sexual partners than his wife.

**Moderator:** are you saying that they really fear their women?

**Female speaker 7:** up to now we have men who haven’t told their women that they were found HIV positive and that they are on ARVs and what we do is to counsel them and inform them about partner notification importance. Another barrier is that besides thinking that they are too busy to test, they end up even convincing their women that the only reason they are not going with them for VCT is that they have no time and women end up believing and supporting the. We keep asking women, where are your husbands and the answer is the same, they are out there looking for money to take care of the family.

**Moderator:** So, you are saying that men have a great influence on women when it comes to accepting that they don’t have time?

**Female Speaker 7:** something like that. Another barrier is self-stigma. Men fear that the community where they reside know their problems while women will to share what is going on in their lives. The last barrier is the one on poor health seeking behavior in general among men. men fear diseases and they believe that if he tests and found positive, that is the end or death has come. This of course goes with lack of information. I guess we can go on and go on repeating what others said but Thank you.

[End of Audio]

Duration: 52 minutes

1. **Groups presentation on strategies to improve uptake of HIV self-testing among men in Kigali, Rwanda**

**Male Speaker** **1**: Strategy one is IEC (Inform, Educate and Communicate) about HIVST. We consider this strategy as the main one or the general one when it comes to messages needed for a health education program for men to improve uptake of HIVST. So, in our group we said that we can use IEC materials to inform people about the use and existence of HIVST kits. One can argue that people don't even know the existence of the kits or where they can get.

**Co-Moderator:** Where do we deliver the messages?

**Male speaker 1:** After seeing that men don't listen to normal channels of communication (radio talk shows, etc.), we can use transport systems or companies to help us deliver the message. In addition of the transport buses, we can use billboards. Another communication channel of help is the church. I believe that except the catholic church that still resist, other churches are okay to deliver messages on HIV prevention. It would be an opportunity to include HIVST messaging in the churches on board.

**Female Speaker 2**: For the barrier number 2, group 2 we found that involving CHWs to deliver messages can be a strong strategy. CHWs can deliver message on HIVST through existing channels like umuganda (monthly communal community work) ibimina (saving groups). These channels can be used to improve uptake of HIVST.

**Male Speaker 1**: How about some men who don't allow CHWs to visit their places because of privacy issues? CHWs are their neighbors and some men believe that their private life can be exposed. Instead we can have a way of delivering such messages normally delivered by CHWs at working places.

**Female Speaker 2**: Us we included that strategy on that barrier on stigma where we suggested that we can place dispenser machines in the working place for people to access easily and privately testing kits.

**Moderator:** How about that barrier on having few places for men to get information related to HIV testing compared to women?

**Female Speaker 2**: Also, on that, in addition to IEC in HFs, we suggested that health care providers in charge of counselling services can be taking the opportunity of talking to men who appear to the hospital as we know that some men decide voluntarily to go test for HIV. so, health care providers can use this opportunity to provide information on self-testing services. Also, each health facility can have a focal point in charge of a dispenser machine to provide testing kits to the men who just come for other health checkups. The cost has just to be affordable.

the dispenser machine strategy will cross cut most of the earlier mentioned barriers like self-stigma and lack of information

**Male Speaker 1**: We can also use the existing condom kiosks in the places close to the bars (hotspots) and also include the self-testing kits

**Female Speaker 2**: I happen to have a lot of information of self-testing, so bear with because I still have some ideas to share. On that one that group on has mentioned on putting the self-testing kits in the existing kiosks , remember that the condoms in there are free of charge, I don't think it is a good idea to make self-testing kits free of charge as we saw condoms being misused at the beginning of the implementation until the number of condoms to provide was limited. So to prevent the misuse of the testing kits, we can limit a small price, would suggest we include HIVST kits in the lodges and bars and hotels where men normally hang out while paying for other services, maybe the cost of the room can include the cost of the kits, I don't know what others think. Or for continuous awareness, we can use hotels, bars and lodges that show football matches to pass the message on HIVST during the break tie. Also, dispenser machines can be providing to these places and place in private areas for men to access the kits.

**Male Speaker 1:** On that idea of including the cost of HIVST kits in the room price, it may be tricky for private industries to do so, instead the dispenser machines would work better.

**Male Speaker 2**: Also, on the health seeking behaviour, we discussed about using married women who visit the health centers and get their HIV statuses to act as messengers or ambassadors to their men. Women can deliver the HIVST kits from HCs to their homes for their men to test after themselves are aware of their results. As we saw that of the barriers is on the trust that their wives’ results are enough, this strategy of using women as ambassadors can cover this behavior. For example, the man will review or be told his wife's result and when handled the kit, he can easily test himself because I the wife turns out to be HIV negative, the man will be motivated to use the kit and test himself as he will be happily waiting for a negative result as his wife was negative. In case of positive result, the man will also be pushed to test or find out his result as way of confirmation or he may think that I have nothing to lose let me test as well. The trust between the husband and the wife will convince the man to take a test after his wife has brought the kit home.

**Moderator:** How about that barrier of men having few options for accessing HIV testing services?

**Male Speaker 1**: Of course, the dispenser machine approach can also cover that one.

**Male Speaker 4:** Do we have focal persons present in organizations who can be delivering health information?

**Female Speaker 2**: Not all the organizations have implemented that and there is no policy present to push them to deliver such information

**Moderator:** PIT (more bias on testing). We mentioned about delivering the message to men who come or show up to the health facilities, how about peer education among men?

**Male Speaker 5:** Yes, having friendly places for men like the ones we provide for youths, youths friendly corners. They youths’ corners really do a great job in terms of awareness at the umudugudu (cell) level

**Female Speaker 2:** But do what we involve in the men corners? (men's attractions are just beer, ladies and football), if we say we put ladies in the corners, it can't be feasible for implementation

**Male Speaker 1:** let’s just say that we will have men friendly places and during the implementation, we can choose feasible approaches. for example, we can use existing government community approaches like car free day and umuganda as channels of communication or have special days for men

**Female Speaker 2:** Adding to that, we can also say that as we normally provide the kits to people who turn out positive during our campaigns of index and partner identification by RBC, the only challenge is that only people who test positive receive the HIVST kits forgetting that some people can be discordant couples. So, we suggest use or including this approach into regular PMTC to provide the kits. Use of already existing structures

**Female Speaker 6:** Use of witnesses (HIV positive) to provide messages of hope or to motivate others to know their statues

**Male Speaker 4:** I am trying to think out loud about a strategy that is usually used for behaviour change in public health …uuuhmmm…Another strategy can Use of incentives as way of behaviour change. But what about the use of incentives as a way of motivation or improving uptake of HIVST among men? We have seen during the fight against malaria, the government using mosquito nets as incentives, and it turned out to be a sustainable approach

**Moderator:** Anhaaa…. like the example of youths getting money 5k as an incentive of those who have managed to take the arts and have suppressed the viral load, I think it is a good idea that can be looked into by the people in charge

**Female Speaker 2:** During car free day, RBC provides services of NCDs screening. We can ask for a special spot to sell or provide the HIVST kits, like choose a pharmacy that will be providing the services

**Female Speaker 7:** Even in the national stadium, when people are watching local football and basketball matches, deliver messages during half time and pass the messages on the screens or have someone designated to deliver the messages. Also, have someone selling the kits and advertise

**Female Speaker 2:** Another strategy is online access or market of the kits like the way KASHA Rwanda is doing by delivering privately condoms and testing kits. We can make sure we increase the online shopping services or providers

**Moderator:** Men have to know that the confidentiality s insured, what do we have to say on that while winding up our discussion?

**Female Speaker 6:** In case of getting a confirmation result after someone has self-tested, men should know that in case they want a confirmation of results, they can visit any clinic of their choice not necessarily the one close to their homes where they believe that care providers know them and may discuss their results anyhow. As care providers, we no longer get or have enough time to provide detailed information on counselling (HIV transmission and prevention). This is a challenge because regarding the myths on HIV, this aspect is not easy to cover. Unless we get more time to explain well to the people

**Moderator:** Thank you once again for your ideas and time. If no one else has a comment or a question, I then welcome Sharon to tell us what is next. Thank you.

**Sharon:** Thank you everyone one again for attending and accepting our invitation. It was a productive workshop and I apologize for not sticking exactly to the time as the agenda shows but I am sure we tried our best as we started at 9:30 instead of 8. Anyway, I want to believe that this discussion with regards to development of HIV self-testing delivery strategies to improve men’s involvement with HIV testing services will yield better result. So, a round of applause for all of us. Thank you. As Moderator said, the room for discussion is still open and we may need to contact you soon if we need more information from anyone of you, kindly collaborate as you did today. Thanks, and enjoy your lunch.

[End of Audio]

Duration: 44 minutes
