# Supplementary material for: Co-creation of a health education program for improving the uptake of HIV self-testing among men in Rwanda: nominal group technique
Source: Heliyon. 2020 Oct 30;6(10):e05378. doi: 10.1016/j.heliyon.2020.e05378 (PMC7610321; doi:10.1016/j.heliyon.2020.e05378)
Supplement: Supplementary File 2 Full contents of HEP [file mmc2.docx]

# Supplementary File 1: Health Education Program for improving men uptake of HIV self-testing

## Contents (1-hour session)

1. Introduction
2. Health locus of control discussion
3. HIV etiology and transmission
4. HIV prevention
5. HIV diagnosis
6. HIV status disclosure
7. HIV / AIDS related stigma and discrimination
8. HIV self-testing

- Background
- Test Procedure

1. HIV care and treatment services
2. References

##

**1.0 Introduction [3 minutes]**

*Men engagement in HIV services in Rwanda is identified as priority in the HIV national strategic plan. Currently, data collected by Rwanda Biomedical Center shows low uptake of HIV services among men in Rwanda.*

In this section introduce the recipient to current state of the HIV/AIDS epidemic in Rwanda briefly.

Discuss HIV national prevalence, UNAIDS 90-90-90 targets and current data for Rwanda. As of 2015 Rwanda demographic and health survey; 24% of men living with HIV/AIDS were unaware of their HIV status.

# **2.0 Health locus of control [5 minutes]**

*Health locus of control is defined as the degree to which people believe that they, as opposed to external forces (beyond their control), have control over their health outcomes. An internal health locus of control suggests that positive health results from one's own doing, willpower or sustained efforts. To assist in improved health seeking behaviour, men have to be educated on taking charge of their health. In this section, the aim is to empower men with knowledge that results in having an internal health locus of control*

Men need to appreciate traditional norms, values, practices and beliefs affect their decision to seek healthcare. It is imperative that men seek knowledgeable about their health status. Discuss economic factors that affect decision to seek health care. In Rwanda, currently there is community-based health insurance for all and a number of public health facilities that offer free HIV testing and counseling.

Discuss cultural and traditional masculine identities and how they affect decision to seek an HIV test.

Discuss perceived susceptibility to disease, the perceived level of severity of the HIV, perceived effectiveness of the preventative behaviour and the costs, which are associated with the preventative behaviour.

# **3.0 HIV etiology and transmission [5 minutes]**

*To better understand the need to obtain an HIV test, men need to have an understanding of what causes HIV infection and how it is transmitted. This is also a good way of slowly delving into the key message for the health education – need to get tested.*

No one knows the origins of AIDS. Some reports suggest that AIDS originated from the United States of America where the first case was reported in 1981 (1). In Rwanda, the first case was reported in 1983 and ever since HIV / AIDS has been a matter of concern in the country and other countries worldwide (2).

The human immunodeficiency virus (HIV) causes the acquired immune deficiency syndrome (AIDS) by damaging the CD4 count or T-cells that protect the immune system of the body. HIV progressively destroys the ability of the body to fight infections and other opportunistic diseases.

HIV cannot survive for a long-time outside human beings (3). It can be transmitted from one person to another through contact of some blood fluids, semen or vaginal fluids, mother-to-child transmission, amongst people who are using the same syringes, and having unprotected sex with patients who are taking ARVs. It is important to remember that HIV cannot be transmitted by shaking hands, sharing food and utensils, and using the same toilets.

Apart from work exposure, people who are at high risk of HIV transmission, are the ones who have unprotected sex, men who have sex with men, drug users who are sharing the same syringes, who are abusing alcohol and not consistently using a condom, who have a sexually transmitted infection; such as syphilis, genital herpes, chlamydia, gonorrhoea, bacterial vaginosis, or trichomoniasis, who have been diagnosed with hepatitis, tuberculosis, or malaria, and who have transactional, and inter-generational sex.

# **4.0 HIV Prevention [5 minutes]**

*To reduce the risk of becoming infected or re-infected by HIV or further spreading the virus to other people, men need to be informed about the modes of HIV transmission, the implications, and how to prevent the further spread. That will encourage them to be tested regularly, practice abstinence, remain faithful to their spouses or partners, consistently use condoms, and refraining from sharing needles.*

HIV / AIDS has no cure. Scientists and researchers are working tirelessly trying to find a vaccine that can prevent HIV infection. Currently there is treatment that ensures you can live with the virus once infected.

The only HIV prevention method that is 100% effective is abstinence from sex. However, there are other multiple HIV prevention methods that have been put in place. Examples include condoms, pre-exposure prophylaxis. These are not 100% effective. There is still a chance of being infected.

# **5.0. HIV Diagnosis [5 minutes]**

*Men need to know the various HIV testing methodologies that are currently at their disposal in Rwanda. While the focus is on improving HIV self-testing uptake; an understanding of what has been traditionally available is important.*

In Rwanda, one can get tested through voluntary HIV counselling and testing (VCT) and provider-initiated HIV testing and counselling (PITC). Periodically, Ministry of Health and partners launch home-based testing using campaigns or surveys. Most testing methods in Rwanda use blood to test for HIV. Results are normally ready withing 30 minutes to 1 hour after blood draw.

# **6.0 HIV Status Disclosure [5 minutes]**

*Men need to be empowered with strategies to disclose their HIV positive status to their sexual partners, family members, employers, and peers. Disclosure is important for preventing and controlling the further spread of HIV, preventing new infections, and treating the currently infected individuals (4). The benefits of disclosure should be emphasized.*

Patients should not be pressured to disclose; they should rather be empowered and prepared for continual counselling support. An important step to disclosure is to disclose your HIV status to your partner.

Discuss good conversation starters for disclosure process to sexual partner such as …“I want to talk with you about something that is important to me” or “I really feel I can trust you and I want to tell you something very personal! Last year I found out that I have HIV. Can I tell you about it?

Discuss importance of disclosure to health care providers, family members for health and social support respectively.

# **7.0 HIV / AIDS related stigma and discrimination [5 minutes]**

*Stigma can be described as an act of identifying, labelling, or attributing undesirable qualities to those people who are perceived as being “shamefully different” and deviant from the social ideal (5). On the other hand, discrimination is perceived as “enacted stigma” (6). Men have to be equipped with knowledge that HIV / AIDS stigma in Sub-Saharan Africa as common and of concern to everyone.*

Some studies suggest that culture is perceived as the base of stigma; since it influences our beliefs, norms, and values that predetermine HIV / AIDS as a negative and immoral act that is not accepted in the society. Men that test positive with HIV/AIDS will be empowered by health care workers to deal with stigma and discrimination. Men need to be empowered and educated about the sources of HIV / AIDS stigma and discrimination, the manner in which individual and societal values impact on HIV / AIDS stigma, and the manifestation on HIV / AIDS stigma and discrimination.

# **8.0 HIV Self testing [10 minutes]**

## 8.1 Background

*HIV self-testing is a relatively new intervention recommended by World Health Organization (7) and Rwanda Ministry of Health (8) as an additional strategy to get more people tested. With this approach, an individual can collect their own specimen (saliva or blood) and test themselves with results interpretation. HIV self-testing has potential to bridge the gap in testing rates among men (9). The barriers listed here were revealed from the first three phases of the study – narrative literature review (10), systematic review (11), in-depth interviews with key stakeholders and a cross sectional survey among male clinic attendees. Discuss each barrier and others that the participant may raise and how HIV self-testing addresses each of these barriers.*

| Number | Barrier | How HIVST addresses barrier. HIVST allows you to… |
| --- | --- | --- |
| 1 | Time (long-waiting time and long queues at HIV testing points) | - Obtain your test kit and test at your own convenience. - There are multiple pharmacies, public health facilities, online platforms offering HIVST kits. |
| 2 | Lack of privacy | - Obtain your test kit and test at your own convenience at a place and time of your choosing. You can test yourself privately. |
| 3 | Health facility too far | - There are multiple pharmacies, public health facilities, online platforms offering HIVST kits. |
| 4 | Confidentiality | - Obtain your test kit and test at your own convenience at a place and time of your choosing. You can test yourself privately. |
| 5 | HIV-related stigma and discrimination | - Obtain your test kit and test at your own convenience at a place and time of your choosing. You can test yourself privately. |
| 6 | Inconvenient hours of operation at testing sites | - There are multiple pharmacies, public health facilities, online platforms offering HIVST kits. |
| 7 | Fear of receiving a positive diagnosis | - Take control of your results. By testing yourself and interpreting you own results, you get to own the result. You have time alone to reflect on the result before seeking professional help and/or social support. |
| 8 | Masculinity (prevents men from expressing emotions in public) | - Obtain your test kit and test at your own convenience at a place and time of your choosing. You can test yourself privately. |
| 9 | Cost | - Currently in Rwanda there are multiple places to obtain HIVST kits free of charge at health facilities and online platforms. |
| 10 | Not knowing where to get tested | - Currently in Rwanda there are multiple places to obtain HIVST kits free of charge at health facilities and online platforms. - Obtain your test kit and test at your own convenience at a place and time of your choosing. You can test yourself privately. |

## 8.2 Test Procedure

OraQuick HIV self-screening kits, manufactured by OraSure Technologies, is the only HIVST kit currently available in Rwanda. The test allows an individual, using an oral swab, to get a result in as little as 20 minutes. OraQuick is the first HIV rapid diagnostic test prequalified by WHO, a status that indicates it complies with international standards.

It is important to walk men through the instructions below on how to do an oral HIV self-screening test. It is also important to emphasize the below:

- Do not eat, drink or use oral care products (such as mouthwash, toothpaste or whitening strips) 30 minutes before starting this test.
- Remove dental products such as dentures or any other products that cover your gums.
- Find a quiet, well lighted place where you can be for at least 20 minutes.
- Always use the directions in the test kit to help read your results correctly.
- If you use glasses to read, you will need them for taking this test.
- Please make sure you have read the information on the back of the outer carton box.
- Make sure you have a timer, watch or something that can time 20 to 40 minutes.
- It may be helpful to have access to a phone to speak directly with a support person. You can dial 114 for support. Someone will speak to you about the rtest procedure, and your results.


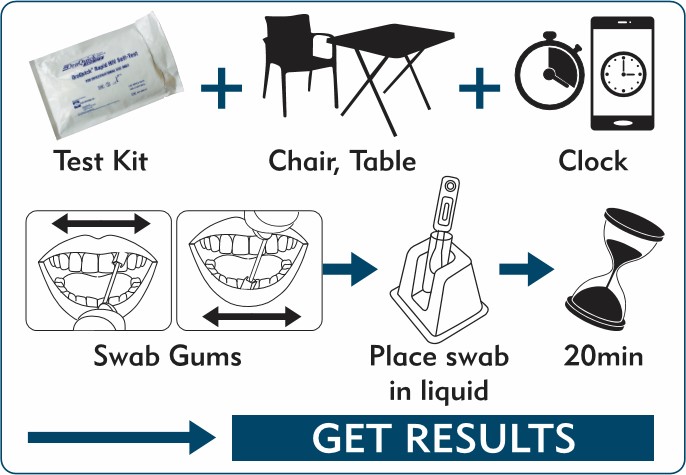


Results interpretation: This has to be emphasized to men accordingly.

- A negative result with this test does not mean that you are definitely not infected with HIV, particularly when exposure may have been within the previous 3 months.
- A positive result with this test does not mean that you are definitely infected with HIV, but rather that additional testing should be done in a medical setting.
- If your test is negative and you engage in activities that put you at risk for HIV on a regular basis, you should test regularly.


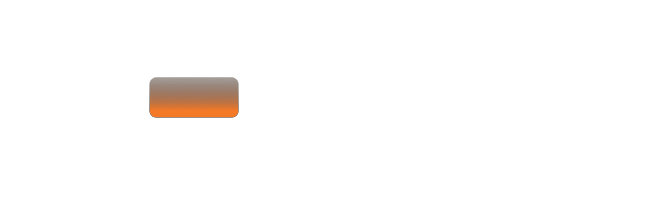

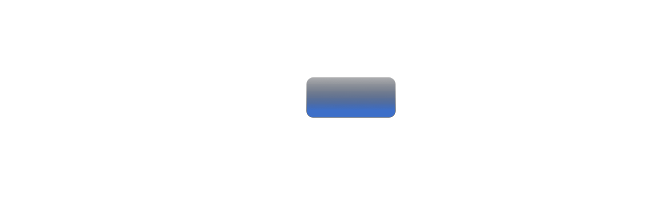

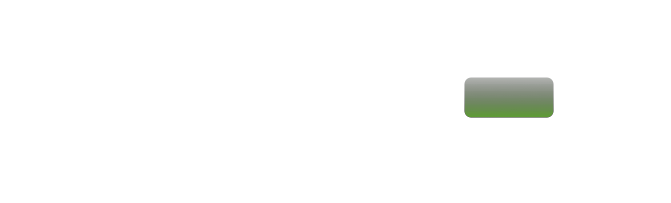


T T T

Positive Negative Unsure

Resul t Resul t Resul t

C

C

C

# **9.0 HIV Care and Treatment Services [5 minutes]**

*With regard to HIV / AIDS treatment, men are required to have knowledge about the benefits of HIV treatment, as well as about the importance of adherence (8). Furthermore, they should familiarise themselves with the most current ARV treatment drugs. The role of these drugs is to interfere with the HIV replication process.*

At health care facility you will receive psychosocial support during counselling by trained personnel. You will discuss the following and more:

- An individual’s understanding of their own diagnosis
- What is ART and who needs ARVs and ART; beliefs and attitudes about ART
- Benefits and challenges of ART and drug resistance
- Importance of ongoing care and regular clinic visits and keeping appointments
- Nutrition
- Safer sex, dual protection, and prevention and treatment of STIs
- Identification of sources of social support (family, treatment supporter, counsellor, support groups, community groups)

# **10.0 References**

1. Nakashima AK, Fleming PL. HIV/AIDS surveillance in the United States, 1981–2001. JAIDS Journal of Acquired Immune Deficiency Syndromes. 2003;32:S68-S85.

2. Cousins S. The past and present violence of Rwanda's HIV epidemic. The Lancet HIV 2019;6(1):e10-e1.

3. Greene W. The molecular biology of human immunodeficiency virus type 1 infection. The New England Journal of Medicine1991;324(5):308-17.

4. Fifield J, O’Sullivan L, Kelvin EA, Mantell JE, Exner T, Ramjee G, et al. Social support and violence-prone relationships as predictors of disclosure of HIV status among newly diagnosed HIV-positive South Africans. AIDS and Behaviour. 2018;22(10):3287-95.

5. Mbonu NC, van den Borne B, De Vries NK. Stigma of people with HIV/AIDS in Sub-Saharan Africa: a literature review. Journal of Tropical Medicine; 2009.

6. Sullivan MC, Rosen AO, Allen A, Benbella D, Camacho G, Cortopassi AC, et al. Falling Short of the First 90: HIV Stigma and HIV Testing Research in the 90–90–90 Era. Springer; 2020.

7. WHO. Guidelines on HIV self-testing and partner notification: supplement to consolidated guidelines on HIV testing services. World Health Organization; 2016. Report No.: 9241549866.

8. Rwanda Biomedical Center. Circular of key changes in HIV management and prevention guidelines. 2018 27 July 2018.

9. Hatzold K, Gudukeya S, Mutseta MN, Chilongosi R, Nalubamba M, Nkhoma C, et al. HIV self‐testing: breaking the barriers to uptake of testing among men and adolescents in sub‐Saharan Africa, experiences from STAR demonstration projects in Malawi, Zambia and Zimbabwe. Journal of the International AIDS Society. 2019;22:e25244.

10. Dzinamarira T, Mashamba-Thompson TP. Factors Contributing Toward Men’s Engagement With HIV Services: A Narrative Review. Journal of Global Health Sciences. 2019;11(10).

11. Dzinamarira T, Kuupiel D, Mashamba-Thompson TP. Health education programs for improving men’s engagement with health services in low-to middle-income countries: a scoping review protocol. Systematic Reviews. 2020;9(1):1-5.
